# Circularly Permuted Far-Red Fluorescent Proteins

**DOI:** 10.3390/bios11110438

**Published:** 2021-11-03

**Authors:** Tianchen Wu, Yu Pang, Hui-wang Ai

**Affiliations:** 1 Department of Molecular Physiology and Biological Physics, and Center for Membrane and Cell Physiology, University of Virginia School of Medicine, 1340 Jefferson Park Avenue, Charlottesville, VA 22908, USA; tw9wa@virginia.edu (T.W.); yp5zr@virginia.edu (Y.P.); 2 Department of Chemistry, University of Virginia, Charlottesville, VA 22908, USA; 3 The UVA Cancer Center, University of Virginia, Charlottesville, VA 22908, USA

**Keywords:** far-red fluorescent protein, circular permutation, genetically encoded fluorescent biosensor, pH sensitivity

## Abstract

The color palette of genetically encoded fluorescent protein indicators (GEFPIs) has expanded rapidly in recent years. GEFPIs with excitation and emission within the “optical window” above 600 nm are expected to be superior in many aspects, such as enhanced tissue penetration, reduced autofluorescence and scattering, and lower phototoxicity. Circular permutation of fluorescent proteins (FPs) is often the first step in the process of developing single-FP-based GEFPIs. This study explored the tolerance of two far-red FPs, mMaroon1 and mCarmine, towards circular permutation. Several initial constructs were built according to previously reported circularly permuted topologies for other FP analogs. Mutagenesis was then performed on these constructs and screened for fluorescent variants. As a result, five circularly permuted far-red FPs (cpFrFPs) with excitation and emission maxima longer than 600 nm were identified. Some displayed appreciable brightness and efficient chromophore maturation. These cpFrFPs variants could be intriguing starting points to further engineer far-red GEFPIs for in vivo tissue imaging.

## 1. Introduction

In the past two and a half decades, the breakthrough in developing genetically encoded fluorescent protein indicators (GEFPIs) has enabled researchers to monitor a plethora of biological activities, such as kinase activities, cellular metabolites, second messengers, membrane potentials, metal ions, redox activities, and neurotransmission [1,2,3,4,5,6,7]. GEFPIs are attractive because they can detect the spatiotemporal dynamics of biological processes in live cells and organisms. Moreover, the nucleic acid sequences of GEFPIs can be incorporated into plasmids, viral vectors, cell lines, and transgenic organisms, enabling transient or long-term expression and convenient distribution across research groups. Furthermore, GEFPIs are compatible with subcellular targeting sequences and tissue-specific promoters, allowing studies on specific subcellular locations or cell types.

GEFPIs typically consist of two essential components: a sensing module that binds or reacts with the target analyte and a fluorescent output module that converts the binding or reaction into a quantifiable signal. The vast majority of GEFPIs are either based on Förster resonance energy transfer (FRET) between a pair of FPs or the modulation of a single FP. To develop single-FP-based indicators, the engineering of circularly permuted FPs (cpFPs), which are topologically mutated FPs, is often the first step, because the fluorescence of fluorescent proteins (FPs) is typically insensitive to fusions at their original N- and C-termini. cpFPs are created by genetically fusing the initial N- and C- termini of FPs through a floppy linker and introducing new termini at another tolerable site within the FP scaffolds. When the sensing modules are fused to cpFPs via the new termini, the conformational changes in the sensing module may readily alter the chromophore protonation state, extinction coefficient, quantum yield, or fluorescence lifetime, resulting in detectable signals [8,9].

To date, most single-FP-based GEFPIs were derived from circularly permuted green or yellow FPs (cpGFPs or cpYFPs) [7]. Recent studies have generated several GEFPIs based on circularly permuted red FPs (cpRFPs) [10,11,12,13]. Because of the advantages of longer-wavelength excitation and emission, there is a strong interest in further expanding the color palette of GEFPIs. First, illuminating biospecimens with long-wavelength light can markedly reduce autofluorescence, scattering, and phototoxicity. Furthermore, the “optical window” for achieving maximal tissue penetration ranges from 600 to 1200 nm wavelength, within which the endogenous absorbers, such as hemoglobin and melanin, present minimal light absorption [14]. Moreover, GEFPIs in the more extended wavelength range are expected to provide additional detection channels, enhancing the capability for multiplexed imaging.

Far-red FPs (FrFPs) and near-infrared FPs (NIFPs) engineered from phycobiliproteins or bacteriophytochromes have greatly expanded the color palette of FPs [15,16,17,18]. These FPs are not structurally homologous to *Aequorea victoria* GFP (avGFP) and do not spontaneously form chromophores within their peptide sequences. Instead, they have to bind a biliverdin cofactor for fluorescence. A recent study performed circular permutation on iRFP, a biliverdin-based NIFP, and discovered several variants exhibiting near-infrared (NIR) fluorescence in *E. coli* and cultured HeLa cells [19]. In other studies, NIR-GECO1 and NIR-GECO2, two GEFPIs for Ca^2+^, were derived by inserting calcium-binding elements into biliverdin-binding mIFP [20,21]. Although biliverdin is present naturally in eukaryotes as a product of heme catabolism, its availability is often limited in various cell types and organisms, leading to diminished brightness [20,21,22,23]. Furthermore, because biliverdin and biliverdin-derived bilirubin are involved in antioxidant and cytoprotective functions [24,25,26], biliverdin-sequestering FPs and GEFPIs may perturb physiology.

FrFPs structurally homologous to avGFP remain as promising options for developing GEFPIs for deep-tissue imaging. In this work, mMaroon1 (NCBI GenBank KX874478) and mCarmine (NCBI GenBank MH062789) [27,28] were selected, two bright FrFPs with excitation and emission maxima above 600 nm, and their tolerance towards circular permutation was examined. mMaroon1 was reported by Bajar et al. in 2016 as one of the brightest monomeric FrFPs with fast chromophore maturation [27]. In addition, when this work began, the excitation of mMaroon1 was most red-shifted (λ_Ex_ = 609 nm) among various available avGFP-like FrFPs. A 630 nm laser or LED light source could excite mMaroon1 efficiently, allowing the combination of mMaroon1 with other FPs for orthogonal four-color fluorescence imaging [27]. Another FrFP utilized in this work, mCarmine, has a peak emission at 675 nm [28]. Compared with mNeptune684 (λ_Em_ = 684 nm) [29], the parental FP of mCarmine, the emission of mCarmine was less red-shifted, but mCarmine maintained a strictly monomeric form while mNeptune684 was found to be in a mixture of dimer and monomer [28]. This study identified and characterized five fluorescent, circularly permuted mutants of mMaroon1 and mCarmine, all of which have peak excitation in the range of 606–610 nm and peak emission in the range of 642–680 nm. These variants are promising starting points for the further development of far-red emitting GEFPIs.

## 2. Materials and Methods

### 2.1. General Materials and Methods

All chemicals were purchased from Fisher Scientific (Hampton, NH, USA), Sigma-Aldrich (St. Louis, MO, USA), Thomas Scientific (Swaysboro, NJ, USA) or VWR (Radnor, PA, USA). DNA oligos were purchased from Integrated DNA Technologies (IDT) (Coralville, IA, USA) or Eurofins Genomics. Restriction enzymes were purchased from Thermo Scientific (Waltham, MA, USA). DNA sequences were analyzed by Eurofins Genomics (Louisville, KY, USA). pcDNA3.1-mMaroon1, pcDNA3-mCarmine were gifts from Michael Lin (Addgene plasmid #83840) and Oliver Griesbeck (Addgene plasmid #109486), respectively. Absorbance and fluorescence measurements were performed with a monochromator-based BioTek Synergy Mx Microplate Reader.

### 2.2. Circular Permutation, Library Construction, and Screening

An overlap polymerase chain reaction (PCR)-based strategy was used to convert mMaroon1 or mCarmine into circularly permuted topologies. Taking cpmMaroon185-186 as an example, two mMaroon1 fragments, including residues 6–186 or residues 185–238, were first separately amplified through two PCRs. The oligonucleotide primers were designed to add an Xho I restriction site to the 5′ end and nucleotides encoding a Gly- and Ser-rich floppy linker to the 3′ end of the 185–238 fragment; and to add nucleotides encoding the Gly- and Ser-rich floppy linker to the 5′ end and a Hind III restriction site to the 3′ end of the 6–186 fragment. Next, the two fragments were assembled in an overlap PCR using the primers containing the Xho I and Hind III restriction sites. The amplified PCR product was digested with Xho I and Hind III and cloned into a compatible pBAD/HisB vector via ligation. The ligation product was used to transform *E. cloni*^®^ 10G competent cells (Lucigen). Cells were allowed to grow on LB agar plates supplemented with 100 μg/mL ampicillin and 0.002% (*w*/*v*) l-arabinose at 37 °C overnight, and then at 4 °C for another 96 h to improve protein folding and chromophore maturation. A customized bacterial colony imaging system, including a Dolan-Jenner Mi-LED Fiber Optic light source, appropriate excitation and emission filters in Thorlabs motorized filter wheels, and a QSI 628 CCD camera, was used to acquire a digital fluorescence image of each plate. Promising clones were selected, and further brightness optimization was performed by conducting successive residue extension or deletion at the newly created N- and C- termini. Following a previous procedure, some mutants were subjected to error-prone (EP)-PCR-based random mutagenesis [30]. Briefly, 50 ng DNA template, 0.5 μM of each primer, 0.1 mM Mn^2+^, 2 μL of an unbalanced dNTP mixture containing 10 mM of three nucleotides and 2.5 mM of the remaining nucleotide, 10 μL of 10× ThermoPol^®^ buffer, and 0.5 μL Taq DNA polymerase (5 U/μL) (NEB M0267S) were included in each 100 μL reaction. The PCR reactions lasted for 38 cycles. Mutagenic PCR products were combined, digested, and ligated into pBAD/HisB. The resultant libraries were screened using the bacterial colony imaging system described above. About 2000 colonies were examined for each library, and 20–50 relatively bright colonies were chosen and used to inoculate liquid cultures for further confirmation. Each culture contained 1 mL 2 × YT supplemented with 100 μg/mL ampicillin and 0.002% (*w*/*v*) L-arabinose. Cells were grown at 37 °C, 250 rpm until the optical density at 600 nm (OD_600_) reached 0.6, and then transferred to 16 °C, 250 rpm for another 48 h. Next, cells were pelleted by centrifugation at 4150× *g* for 10 min and lysed with 300 μL of B-PER bacterial protein extraction reagents (Pierce). The fluorescent intensity of each cell lysate was examined using a BioTek Synergy Mx Microplate Reader.

### 2.3. Protein Expression and Purification

pBAD plasmids harboring the cpFP variant genes were used to transform *E. cloni*^®^ 10G competent cells (Lucigen), which were next plated on 2 × YT agar plates supplemented with 100 μg/mL ampicillin and 0.002% (*w*/*v*) L-arabinose. The plates were incubated at 37 °C overnight and then at room temperature for another 24 h. A single colony was isolated and inoculated in 5 mL of liquid 2 × YT medium supplemented with 100 μg/mL ampicillin. The starter culture was shaken at 250 rpm and 37 °C overnight, then diluted 100-fold into 500 mL 2× YT supplemented with 100 μg/mL ampicillin. Protein expression was induced by adding a final concentration of 0.2% (*w*/*v*) L-arabinose at OD_600_ of ~0.6. Next, the culture was moved to 16 °C and 250 rpm for another 48 h. Bacterial cells were harvested by centrifugation at 4150× *g* for 20 min. Cells were resuspended in 30 mM Tris-HCl buffer (pH 7.4) and lysed by sonication. Cell debris was removed by centrifugation at 12,500× *g* for 30 min at 4 °C. The clear supernatant was applied to Ni-NTA agarose beads (Pierce), and His6-tagged proteins were purified according to the manufacturer’s instructions. The eluted proteins were verified with sodium dodecyl sulfate-polyacrylamide gel electrophoresis (SDS-PAGE) to assure at least 95% purity. The proteins were further concentrated using Amicon Ultra Centrifugal Filter Units (10,000 Da molecular weight cutoff).

### 2.4. In Vitro Characterization

A BioTek Synergy Mx Microplate Reader was used to perform all measurements. Protein concentrations were determined by measuring the absorbance following alkaline denaturation and assuming extinction coefficient (*ε*) = 44,000 M^−1^ cm^−1^ at 446 nm [31,32], or by using a Pierce^TM^ 660 nm Protein Assay Kit (Thermo Scientific, Waltham, MA, USA) and a series of bovine serum albumin (BSA) standards with known concentrations. Two extinction coefficient values were then calculated for each protein by dividing the peak absorbance of the undenatured protein by the product of the pathlength and the protein concentration determined from either the alkaline denaturation method or the Pierce^TM^ 660 nm Assay [33,34].

To record absorbance, proteins were diluted using 30 mM Tris-HCl (pH 7.4) to a final concentration of 1 μM based on the alkali denaturation method, and the monochromator scanned from 450 to 750 nm. To record excitation spectra, the emission wavelength was fixed at 650 nm, and the excitation monochromator scanned from 450 to 630 nm. To record emission spectra, the excitation wavelength was fixed at 590 nm, and the emission monochromator scanned from 610 to 800 nm.

To determine quantum yields, mMaroon1 was used as the reference (*Φ* = 0.11) [27]. Briefly, each protein was diluted to a series of concentrations in 30 mM Tris-HCl (pH 7.4). Fluorescent emission spectra were recorded for each sample with a 1 nm interval. The overall fluorescent intensity was integrated by adding all values across emission wavelengths. Next, the integrated fluorescent intensity and the absorbance of each sample were plotted as y and x. A linear relationship was assumed to calculate the slope (S) of the function. The quantum yield of the protein was then determined from *Φ*_protein_ = *Φ*_reference_ × (S_protein/_S_reference_). To characterize the oligomeric status of the proteins, the eluates from Ni-NTA affinity purification were further injected into a HiLoad 16/60 Superdex 200 pg size exclusion column (GE Healthcare). A 30 mM Tris-HCl (pH 7.4) buffer was used for elution. To determine the apparent *p*K_a_, a series of buffers containing 200 mM citric acid and 200 mM boric acid with pH values ranging from 3 to 10 were prepared. The pH titration was performed by mixing 99 μL of the buffers described above with 1 μL of each protein, and the final protein concentration was 100 nM. The fluorescence of each sample was determined. The values were plotted as a function of pH values and used to fit the Hill equation.

## 3. Results

### 3.1. Circular Permutation of mMaroon1 and mCarmine

**Figure 1:**
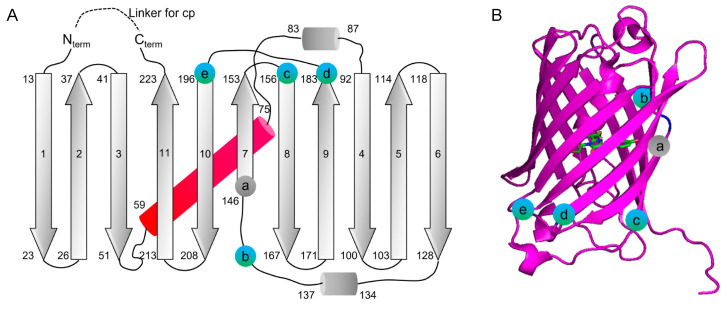
Location of mMaroon1 circular permutation sites explored in this study. (**A**) Unsuccessful and successful circular permutation sites are labeled as grey and blue spheres, respectively, on the secondary structure elements of mMaroon1. Residue numbers for the start and end of secondary structure elements are based on an mMaroon1 structure predicted by SWISS-MODEL using mCardinal (PDB: 4OQW) as the template. Cylinders represent α-helices and arrows represent β-strands. β-strands are labeled with consecutive numbers, indicating their positions in the secondary structure. Grey or blue circle labels the first residue of each circular permutation construct: (a) cpmMaroon145-148, (b) cpmMaroon141-152, (c) cpmMaroon158-152, (d) cpmMaroon185-186, (e) cpmMaroon196-186. (**B**) Cartoon representation of the structure of mMaroon1 predicted by SWISS-MODEL using mCardinal (PDB: 4OQW) with positions of circular permutation denoted in (**A**). The chromophore is presented in sticks mode.

**Figure 2:**
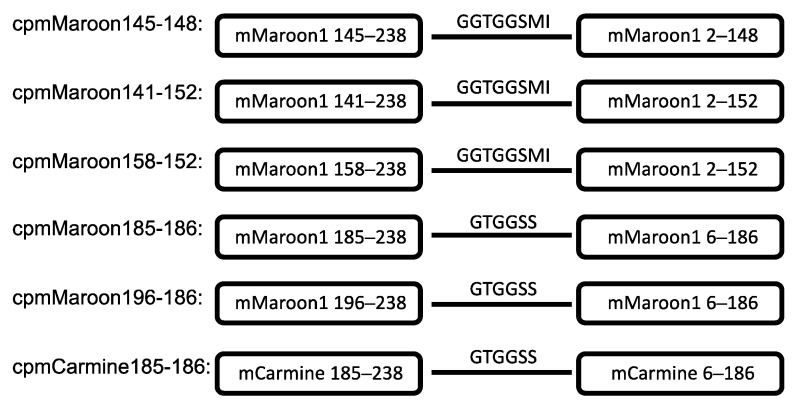
Primary structures of circularly permuted mMaroon1 and mCarmine variants. The portion in the circled bars represents the primary sequence of mMaroon1 or mCarmine. The original N- and C-termini are linked by an eight-residue “GGTGGSMI” linker or a six-residue “GTGGSS” linker.

**Figure 3:**
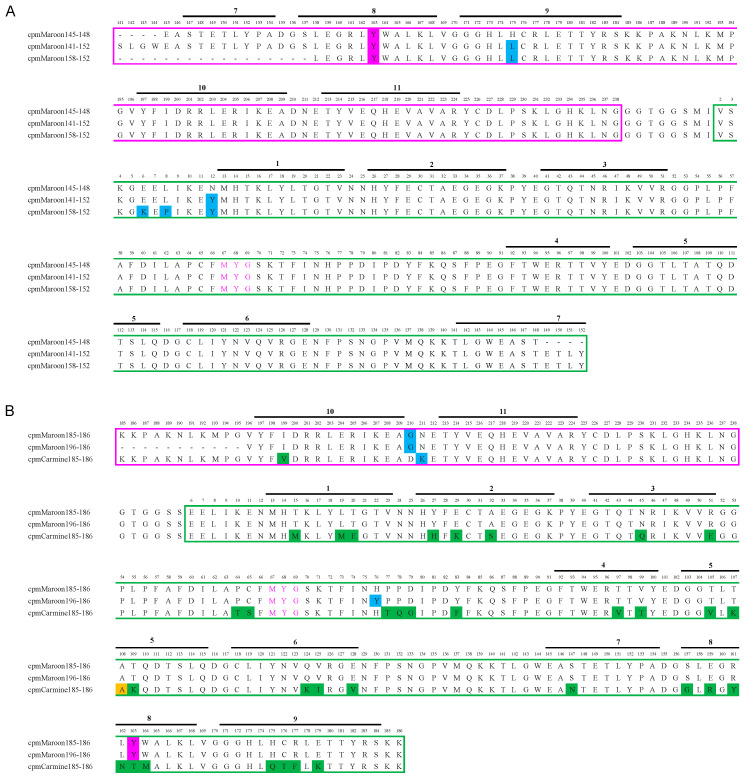
Sequence alignment of circularly permuted mMaroon1 and mCarmine variants, including (**A**) cpmMaroon145-148, cpmMaroon141-152, and cpmMaroon158-152; and (**B**) cpmMaroon185-186, cpmMaroon196-186, and cpmCarmine185-186. The sequences derived from mMaroon1 or mCarmine are in magenta and green boxes. Individual β-strands are denoted by the black lines and numbers above the sequences. The chromophore-forming residues (residues 67–69) are shown in magenta fonts. The mutations from random mutagenesis are shaded in blue. The differences between the original mMaroon1 and mCarmine sequences are shaded in green. One residue in cpCamrmine185-186, initially different from mMaroon1, was mutated to the same residue as mMaroon1 during random mutagenesis, and this residue is shaded in yellow. The D163Y mutation (shaded in magenta) was initially discovered during the engineering of cpmMaroon185-186. Because it was transferable for enhancing the brightness of mMaroon1, it was introduced into all circularly permuted mMaroon1 variants.

The circular permutation sites that were examined are shown in Figure 1, which were drawn based on a structure predicted by SWISS-MODEL using mCardinal (PDB: 4OQW) as the template [35,36]. This paper refers to circularly permuted variants of mMaroon1 and mCarimine as cpmMaroon and cpmCarmine, respectively, followed by two numbers indicating the starting and ending residues numbered according to the mMaroon1 sequence (Figure 2 and Figure 3).

This study started with a site in β-strand 7 of mMaroon1 because this position has been utilized most widely to circularly permutate avGFP and avGFP-like proteins for GEFPI development [3,37,38,39]. This circular permutation site, identified as residue 146 in β-strand 7 in mMaroon1, structurally aligns with residue 145 in avGFP. A fusion protein was generated between residues 145–238 and residues 6–148 of mMaroon1 connected through a six-residue “GTGGSS” floppy linker. Unfortunately, the construct did not exhibit detectable fluorescence even after incubation at 4 °C for 96 h. To exclude the possibility that a too short floppy linker might impact protein folding, the linker was extended by fusing residues 145–238 and 2–148 of mMaroon1 through an eight-residue linker. In addition, a D163Y mutation was introduced, which was identified as enhancing the brightness of mMaroon1 and several other circularly permuted variants (discussed below). The resulting construct was termed cpmMaroon145-148 (Figure 2), but unfortunately, it was still nonfluorescent. EP-PCRs were performed to randomize cpmMaroon145-148 but did not yield any fluorescent variants either.

Next, the N- and C- termini of cpmMaroon145-148 were extended by adding the original residues in mMaroon1 with the hope of rescuing the fluorescence. One amino acid was extended at each terminus every time, and the fluorescence of these variants was examined. The variant having four amino acid extensions on both termini showed moderate far-red fluorescence. This variant was subjected to one round of EP-PCR random mutagenesis, and another variant with improved brightness was identified and named cpmMaroon141-152 (Figure 2 and Figure 3A). DNA sequencing confirmed that cpmMaroon141-152 gained two mutations (H175L and N12Y) through the random mutagenesis and library screening process.

To identify residues at the N- and C- termini of cpmMaroon141-152 essential for fluorescence, further truncation with cpmMaroon141-152 was performed. Two residues were removed from the N-terminus each time and the fluorescence of these variants was examined. A variant with as many as 17 residues truncated from the N-terminus showed detectable far-red fluorescence. An additional round of EP-PCR was performed to improve brightness further. A variant with bright fluorescence was obtained and termed cpmMaroon158-152 (Figure 2). Compared with cpmMaroon141-152, cpmMaroon158-152 gained two additional unique mutations (E6K and L8P) from random mutagenesis (Figure 3A). Unlike the N-terminal truncations, removing even one residue from the C-terminus of cpmMaroon141-152 led to undetectable fluorescence.

Meanwhile, circular permutation was also attempted at a location between β-strands 9 and 10. This location has been successfully used to create the circularly permuted variants of mCherry and mKate [33,40]. Moreover, voltage indicators based on circularly permuted mKate between β-strands 9 and 10 have been reported [41]. Thus, a similar variant of mMaroon1 was created. The initial construct with circular permutation at residue 185 was barely fluorescent. One round of random mutagenesis was performed and a mutant, termed cpmMaroon185-186, with bright fluorescence (Figure 2 and Figure 3B) was discovered. cpmMaroon185-186 carried two mutations (D163Y and D210G) different from the initial circular permutation construct.

A truncation strategy was again applied to cpmMaroon185-186 to find the terminal residues indispensable for fluorescence. Fluorescence was not observed when the 12th residue was deleted from the N- terminus of cpmMaroon185-186. Next, the mutant, with 11 amino acid residues truncated from the N-terminus, was subjected to random mutagenesis. One variant, with moderate far-red fluorescence, was selected and termed cpmMaroon196-186 (Figure 2 and Figure 3B). cpmMaroon196-186 gained one mutation (H76Y) from the random mutagenesis process.

After successfully developing the cpmMaroon variants, circular permutation on mCarmine at residues 145 and 185 was next explored. Unfortunately, cpmCarmine145-148 did not show any observable fluorescence. The initial construct of cpmCarmine185-186 displayed relatively dim fluorescence and was subjected to random mutagenesis. After library screening, a brighter variant (Figure 3B) with two mutations (V108A and N211K) was identified.

### 3.2. Characterization of Circularly Permuted mMaroon1 and mCarmine Variants

**Figure 4:**
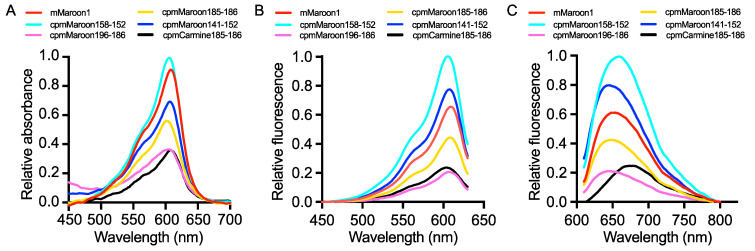
Absorbance, excitation, and emission spectra for circularly permuted mMaroon1 and mCarmine variants. (**A**) Absorbance spectra in order of highest to lowest intensity at 600 nm: cpmMaroon158-152 (cyan), mMaroon1 (red; included for comparison), cpmMaroon141-152 (blue), cpmMaroon185-186 (yellow), cpmCarmine185-186 (black), and cpmMaroon196-186 (magenta). (**B**) Excitation spectra labeled in the same color schedule as in panel A. (**C**) Emission spectra labeled in the same color schedule as in panel A.

The five fluorescent variants in *E. coli* were expressed and the proteins prepared via Ni-NTA affinity purification. First, the key biophysical parameters of each protein were quantified, including absorption spectrum (Figure 4A), excitation spectrum (Figure 4B), emission spectrum (Figure 4C), quantum yield (*Φ*), and extinction coefficient (*ε*) (Table 1). Two different methods, alkaline denaturation [31,32] and Pierce™ 660 nm Protein Assay, were used to quantify the concentrations of each protein. The former method uses the base to denature proteins, resulting in exposed chromophores with a known extinction coefficient of 44,000 M^−1^ cm^−1^ at ~446 nm [31,32], so it only measures the concentrations of the protein portions with a mature chromophore. In contrast, the latter quantifies the total proteins with and without a mature chromophore. Thus, the former method often leads to a smaller protein concentration and a higher extinction coefficient. The relative protein folding and chromophore maturation efficiency (Table 1) can then be calculated from the extinction coefficient or brightness values determined via the two protein quantification methods [33,34].

Among these circularly permuted variants, cpmMaroon158-152 showed the highest brightness and folding/chromophore maturation efficiency, close to the parental mMaroon1 protein. Another variant, cpmMaroon141-152, displayed a high quantum yield, but its folding/chromophore maturation efficiency was ~2.5× lower than cpmMaroon158-152. In addition, the brightness and folding/chromophore maturation efficiency of the other three variants, cpmMaroon185-186, cpmMaroon196-186, and cpmCarmine185-186, were significantly reduced. In particular, cpmMaroon196-186 displayed the lowest chromophore maturation efficiency, and the deletion of the loop between β-strands 9 and 10 reduced the folding/chromophore maturation efficiency from 9% to 5%.

All variants showed far-red fluorescence inherited from their parental FPs with peak excitation wavelengths between 606–610 nm (Table 1). However, the peak emission varied among different variants. cpmCarmine185-186 exhibited the most far-red shifted peak emission at 680 nm, followed by cpmMaroon158-152 showing peak emission at 660 nm. The rest of the variants presented a slightly blue-shifted peak emission ranging from 642–648 nm.

**Figure 5:**
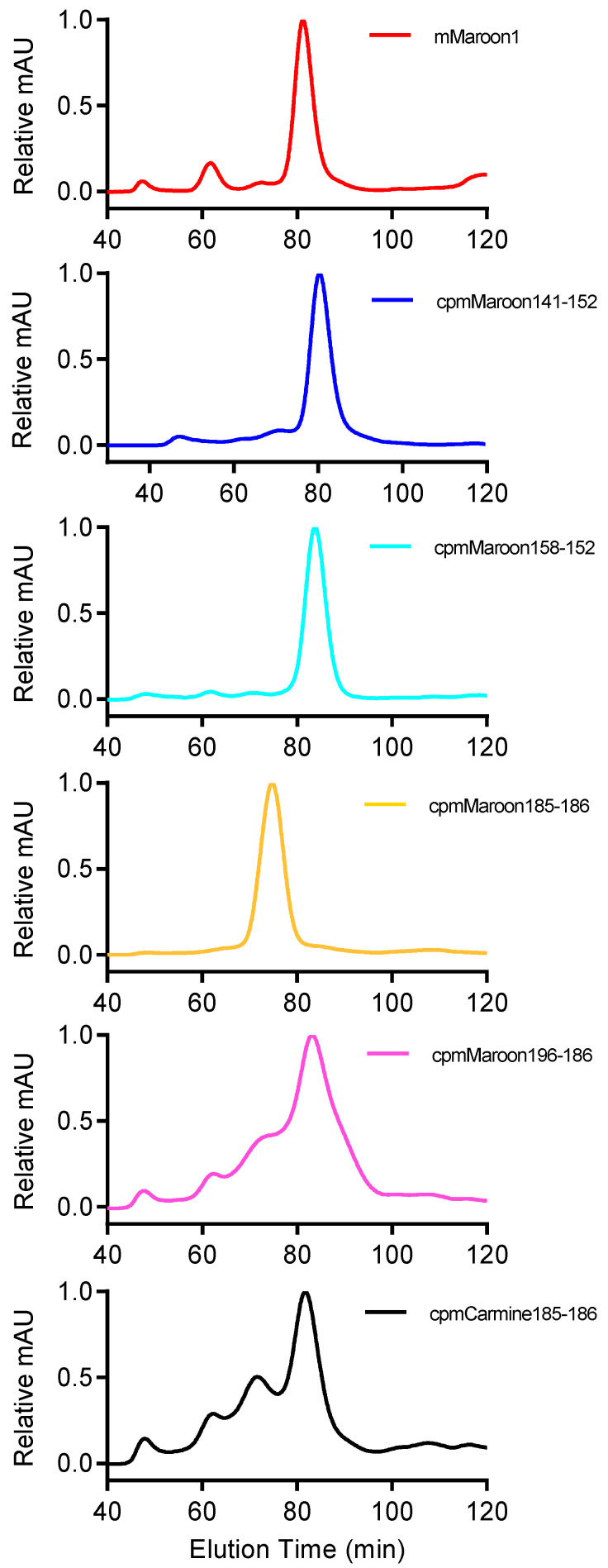
Gel filtration chromatography elution profiles of different variants. The absorbance detection was at 280 nm. The graphs from upper to lower are for mMaroon1, cpmMaroon158-152, cpmMaroon196-186, cpmMaroon185-186, cpmMaroon141-152 and cpmCarmine185-186.

Gel filtration chromatography was performed to examine the oligomerization states of these proteins (Figure 5). Under our conditions, mMaroon1, cpmMaroon141-152, and cpmMaroon158-152 were mostly monomers, while most of cpmMaroon185-186 adopted a dimeric structure. Furthermore, cpmMaroon196-186 and cpmCarmine185-186 displayed multiple oligomeric states, with ~70% of cpmMaroon196-186 and ~60% of cpmCarmine185-186 being monomeric.

**Figure 6:**
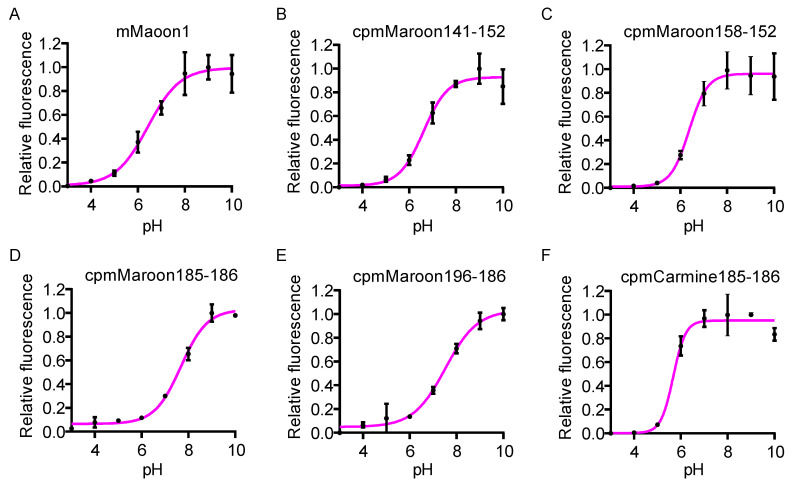
pH dependency of the fluorescence of different variants. Data are presented as mean ±s.d. of three technical replicates. Magenta lines are the fitting of the data with the Hill equation, and apparent *p*K_a_ values (defined as the pH causing 50% of the overall fluorescence intensity change) are derived and presented in Table 1.

Finally, the pH dependency of these FP variants in terms of fluorescence intensity was examined (Figure 6). The Hill equation was used to fit the data and derive the apparent *p*K_a_ values. cpmMaroon185-186 and cpmMaroon196-186 were very sensitive to pH variations around the physiological pH (7.4) and presented high *p*K_a_ values (7.7 and 7.5, respectively), while the *p*K_a_ values of cpmMaroon141-152 and cpmMaroon158-152 were close to that of mMaroon1, in the range of 6.4–6.6. cpmCarmine185-186 had the lowest *p*K_a_ value of 5.6 and was insensitive to pH changes around pH 7.4.

## 4. Discussion

In this work, five fluorescent, circularly permuted variants of mMaroon1 and mCarmine were identified. Among them, cpmMaroon158-152 displayed the greatest fluorescence intensity and the best chromophore folding efficiency. With an impaired chromophore maturation compared with cpmMaroon158-152, cpmMaroon141-152 still showed good fluorescence intensity. Both of these variants carried circular permutation sites between β-strands 7 and 8. In addition, cpmMaroon and cpmCarmine variants were generated with circular permutation sites between β-strands 9 and 10, but there was a significant decline in their fluorescence intensity and chromophore maturation. Therefore, the loop between β-strands 7 and 8 seemed to be more tolerant of introducing new N- and C- termini than the loop between β-strands 9 and 10.

Circular permutation within β-strand 7 is recognized as the most classic circular permutation site, leading to the common applications of cpFPs to developing various single-FP-based GEFPIs [3,10,37,42]. The introduction of new termini within β-strand 7 of mMaroon1 or mCarmine completely abolished the far-red fluorescence. It is highly possible that circular permutation at this site may perturb the environment required for far-red chromophore formation and stabilization, causing the loss of fluorescence. However, recently, a preprint appeared reporting the development of several far-red fluorescent calcium indicators with excitation and emission maxima at ~596 nm and ~644 nm, respectively, based on circular permutation of another FrFP, mKelly2, within β-strand 7 [43]. Thus, it may still be possible to rescue the fluorescence of cpmMaroon145-148 and cpmCarmine145-148 through further protein engineering efforts.

Five fluorescent variants were identified bearing circular permutation sites between β-strands 7 and 8 or β-strands 9 and 10. cpmMaroon141-152 with four residues extended on each of the N- and C- termini of cpmMaroon145-148 displayed a relatively strong fluorescence. The engineering of cpmMaroon158-152 further verified the essential role of the four residues at the newly created C- terminus in proper protein folding. Furthermore, N12Y and H175L mutations in both cpmMaroon141-152 and cpmMaroon158-152, in addition to E6K and L8P in cpmMaroon158-152, might contribute to the enhanced brightness of these variants by improving protein folding and chromophore maturation.

Initially, the variant cpmMaroon185-186 did not display bright fluorescence, until an unexpected key mutation (D163Y) was acquired through random mutagenesis. In fact, the brightness of *E. coli* expressing mMaroon1 was also enhanced after incorporating the D163Y mutation (data not shown). Therefore, the mutation was introduced into all circularly permuted mMaroon1 mutants examined in this work. Unfortunately, it was still unable to rescue the fluorescence of cpmMaron145-148. Deleting the loop between β-strands 9 and 10 of cpmMaroon185-186 led to cpmMaroon196-186, which contained another key mutation (H76Y) from random mutagenesis to remarkably rescue the fluorescence. However, the introduction of H76Y back to mMaroon1 did not cause an improvement in brightness (data not shown).

cpmCarmine185-186 gained two mutations (N211K and V108A) through random mutagenesis. Interestingly, the V108 in mCarmine mutated to A, which was identical to this residue in other cpmMaroon variants, suggesting that this non-surface residue may play a crucial role in stabilizing these cpFrFPs.

Further improvements of biophysical properties of these cpFrFP variants, including their brightness, maturation speed, and photostability, may be achieved by additional rounds of directed evolution. In addition, selecting far-red emitting FPs different from mMaroon1 and mCarmine for circular permutation may be another possibility to obtain cpFrFP variants with improved fluorescence properties. In particular, mMaroon1 has been reported to show some dimerization propensity [28], which may increase the difficulty in obtaining successful circular permutation at sites near the assumed dimer interacting interface.

For prospects, the most direct and feasible application for the cpFrFP variants would be the construction of single-FP-based GEFPIs. Although these circular permutation sites were different from the classic site within β-strand 7, the fluorescence of the cpFrFP variants was sensitive to amino acid changes in their N- and C- termini. In particular, it appears promising to fuse sensory domains to the new termini between β-strands 9 and 10, since there are precedents for creating successful voltage indicators [41]. Moreover, the cpFrFP variants are desirable since they have excitation and emission peaks above 600 nm. It is thus possible to use 630 nm laser or LED light sources to achieve good imaging depth in tissue with one-photon fluorescence microscope setups.

Unpublished results in the authors’ own lab support the feasibility of using cpmMaroon185-186 and cpmMaroon196-186 to develop single-FP-based GEFPIs. The pH-dependency of the fluorescence of these cpFrFP variants was determined by the authors. cpmMaroon185-186 and cpmMaroon196-186 have apparent *p*K_a_ values close to the physiological pH (7.4). Since most single-FP-based indicators rely on the modulation of chromophore ionization by the fused sensory domains, cpFPs with *p*K_a_ values near the physiological pH would be favorable for developing single-FP-based GEFPIs.

Although the primary purpose of this research was to develop cpFrFPs for constructing single-FP-based indicators, these cpFrFP variants may find applications in developing bimolecular fluorescence complementation (BiFC) assays, which use two complementary fragments of an FP to visualize protein–protein interactions in living cells [44]. The circular permutation sites explored in this research may serve as FP split sites. The spectral separation of these variants from orange FPs and biliverdin-based NIFPs makes them attractive when performing multicolor assays. Furthermore, these cpFrFP variants could serve as donors or acceptors in Förster resonance energy transfer (FRET) experiments. These circularly permuted variants allow the exploration of new dipole orientations relative to the donor, to which the FRET efficiency is very sensitive [9]. Because of the low quantum yields of these cpFrFP variants, they likely will not generate much sensitized emission when being used as FRET acceptors, but may still be useful for fluorescence-lifetime-based measurements [45]. Moreover, some cpFrFP variants, such as cpmMaroon185-186 and cpmMaroon196-186, may potentially be utilized as far-red fluorescent pH indicators for monitoring pH changes in cellular compartments or activity-dependent exocytosis [46,47,48].

In summary, this work has resulted in five fluorescent circularly permuted variants of mMaroon1 and mCarmine, all with peak excitation and emission longer than 600 nm. The authors are optimistic that a continued improvement of the cpFrFP variants described in this work would lead to brighter, more stable, better folded, and less oligomerized variants, which will be even more valuable for developing single FP-based GEFPIs, as well as BiFC- and FRET-based assays.

## Figures and Tables

**Table 1:** Properties of circularly permuted mMaroon1 and mCarmine variants.

FrFP Variant	λ_Ex_ (nm)	λ_Em_ (nm)	*p*K_a_	*Φ*	Alkaline Denaturation			Pierce™ 660 nm Protein Assay			Folding and Chromophore Maturation Efficiency ^§^ (%)
					*Ε*^‡^ (M^−1^ cm^−1^)	Brightness ^†^		*Ε*^‡^ (M^−1^ cm^−1^)	Brightness ^†^		
						(mM^−1^ cm^−1^)	(%)		(mM^−1^ cm^−1^)	(%)	
mMaroon1	610	658	6.4	0.11	70,616	7.8	100	31,977	3.5	100	45
cpmMaroon141-152	606	642	6.6	0.14	64,507	9.0	116	9954	1.4	40	15
cpmMaroon158-152	606	660	6.4	0.11	86,667	9.5	123	32,624	3.6	103	37
cpmMaroon185-186	610	648	7.7	0.11	54,018	5.9	76	4705	0.5	15	9
cpmMaroon196-186	608	642	7.5	0.09	29,792	2.7	35	1574	0.1	3	5
cpmCarmine185-186	606	680	5.7	0.07	44,457	3.1	40	6340	0.4	11	14

^‡^ Determined separately using alkaline denaturation or Pierce™ 660 nm Protein Assay. ^†^ Defined as the product of *Φ* and *ε*. ^§^ Defined as the quotient of the brightness results from the two methods (value of Pierce™ 660 nm Protein Assay divided by the value of alkaline denaturation method).

## Data Availability

The original data are available upon request from the corresponding author.
